# Phase-transition assisted mechanical behavior of TiZrHfTa_*x*_ high-entropy alloys

**DOI:** 10.1038/s41598-018-30892-x

**Published:** 2018-08-22

**Authors:** Shuo Huang, Wei Li, Erik Holmström, Levente Vitos

**Affiliations:** 1 Applied Materials Physics, Department of Materials Science and Engineering, Royal Institute of Technology, Stockholm, SE-100 44 Sweden; 2 Sandvik Coromant R&D, 126 80 Stockholm, Sweden; 3 Department of Physics and Astronomy, Division of Materials Theory, Uppsala University, SE-75120 Uppsala, Sweden; 4 Institute for Solid State Physics and Optics, Wigner Research Centre for Physics, H-1525 Budapest, Hungary

## Abstract

Recent developments of high-entropy alloys with high strength and high ductility draw attention to the metastability-engineering strategy. Using first-principle theory, here we demonstrate that reducing the Ta level in the refractory TiZrHfTa_*x*_ system destabilizes the body-centered cubic (bcc) phase and leads to the appearance of the hexagonal close-packed (hcp) phase embedded in the bcc matrix. The alloying-induced features of the elastic parameters for the cubic and hexagonal structures are mapped out in details, and strong sensitivity to the crystal lattice and chemistry is revealed. Results show softening of the bcc matrix with decreasing Ta concentration which ensures ductile behavior. However, the elastically nearly isotropic hcp precipitates possess enhanced resistance against shear which promotes strengthening of the TiZrHfTa_*x*_ dual-phase system. The present atomic-level insight provides strong evidence to the experimental observation, and emphasizes the significance of quantum-design for advanced multi-phase high-entropy alloys with excellent strength-ductility combinations.

## Introduction

The development of metallic materials with high strength and at the same time enhanced ductility has never faded for scientific interests and technological applications. Over the past years, high-entropy alloys (HEAs) have drawn significant attention as they open up a near-infinite compositional space for designing materials with exceptional properties^1–10^. In general, HEAs are composed of multi-principal elements with equal or near-equal molar ratios, and most of them show preference to stabilize in simple solid-solution phases with face-centered cubic (fcc), body-centered cubic (bcc), or hexagonal close-packed (hcp) underlying lattices^11^. Particularly, the bcc HEAs based on refractory elements usually possess good phase stability and mechanical strength^12–14^, which makes them potential high-temperature structural materials. However, the low ductility observed for several typical system (e.g., NbMoTaW shows compressive plasticity of ~2% at room temperature^12^) hinders their applications.

Recently, a metastable transformation-induced plasticity effect was introduced in the case of HEAs^15–18^. These alloys consist of multiple phases (i.e., fcc and hcp phases), and show improved strength and ductility as compared to many single-phase HEAs. Similar phenomenon was revealed in the refractory TiZrHfTa_*x*_ HEAs that consist of bcc and hcp phases^19^. Understanding the intrinsic elastic properties of individual structures is critical for optimizing the mechanical performance of such dual-phase HEAs. Unfortunately, today very limited information is available for HEAs with hcp structure, which can be attributed to the complexity of the problem related to the chemical and magnetic disorder in connection with the multicomponent nature of the HEAs. In this work, we employ first-principle theory to bring to light the elastic behavior of the refractory TiZrHfTa_*x*_ HEAs adopting bcc single-phase or bcc/hcp dual-phase with varying Ta fraction from 0 to 2.

## Results and Discussion

**Figure 1:**
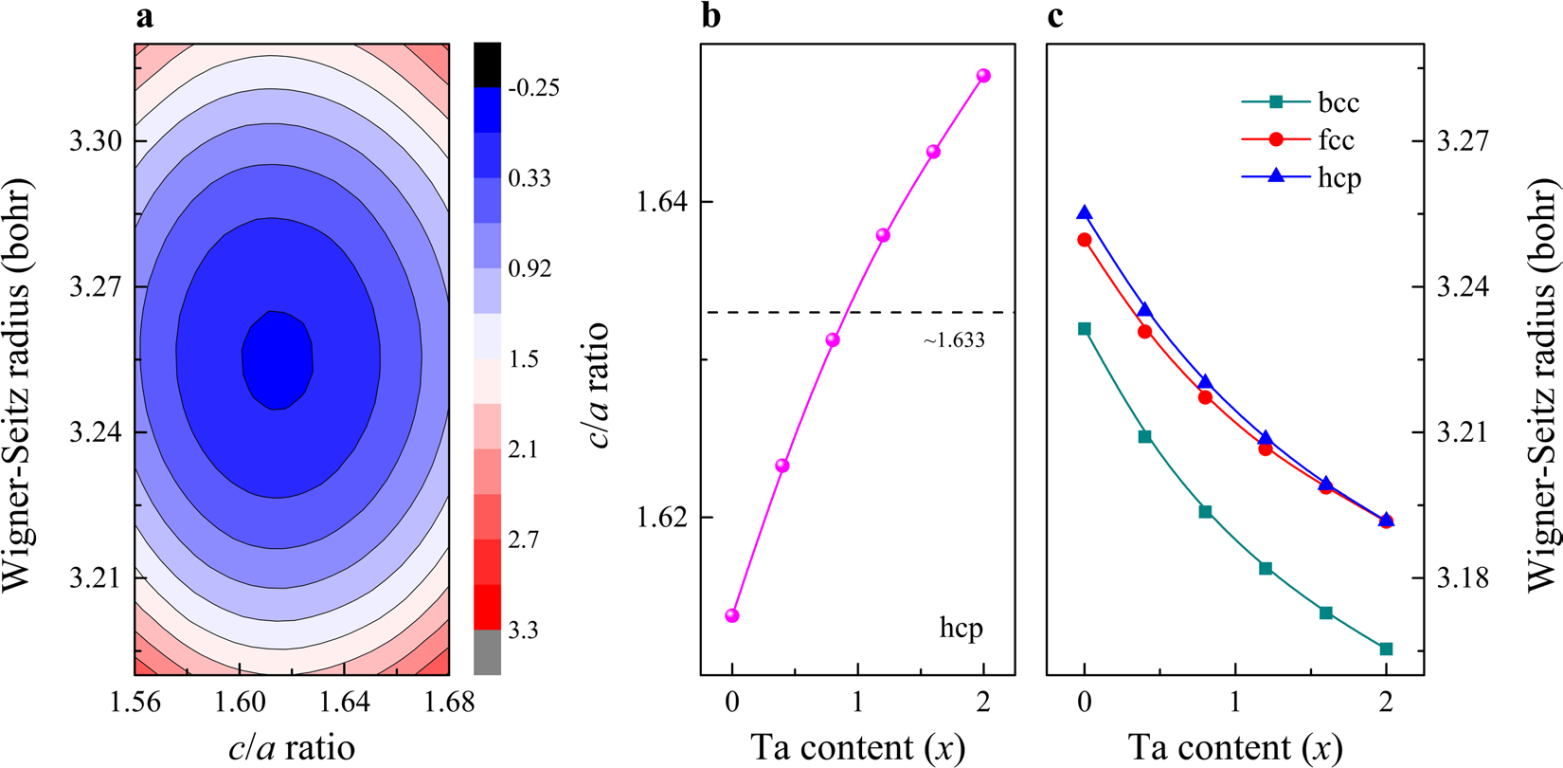
(**a**) The map of total energy (units of mRy) for the hcp phase of TiZrHf as a function of the Wigner-Seitz radius *w* and the hexagonal axial ratio *c*/*a*. The energies are plotted relative to the energy minimum. (**b**) The optimized *c*/*a* ratio for the hcp phase of TiZrHfTa_*x*_ as a function of composition. (**c**) The equilibrium *w* for the bcc, fcc and hcp phases of TiZrHfTa_*x*_ as a function of composition.

It is known that the refractory elements Ti, Zr and Hf are stable in the hcp phase at ambient conditions. In Fig. 1a, we present the total energy of TiZrHf for the hcp structure as a function of the Wigner-Seitz radius *w* and the hexagonal axial ratio *c*/*a*. There is a local minimum in the configurational space with *w* and *c*/*a* values around 3.255 bohr and 1.614, respectively. These optimized *w* and *c*/*a* values are slightly larger than 3.234 bohr and 1.587 determined from the linear rule of mixture based on the corresponding experimental data of the alloy components^20^. Extending this study to TiZrHfTa_*x*_ (Fig. 1b,c) shows that Ta addition gradually decreases *w* and increases *c*/*a* of the host alloy. This trend can be attributed to the smaller atomic radius of the bcc stabilizer elemental Ta as compared to those of the other components. Regarding the Ta concentration dependence of the volume, in Fig. 1c, we compare the present hcp volumes with those obtained using bcc and fcc underlying lattices. It is found that the volume of the bcc phase is always lower than that of the hcp phase irrespective of the composition. We notice that the denser bcc lattice is in line with the experimentally observed volume collapse (observed, e.g., for Zr) at the hcp to bcc phase transition^21^.

**Figure 2:**
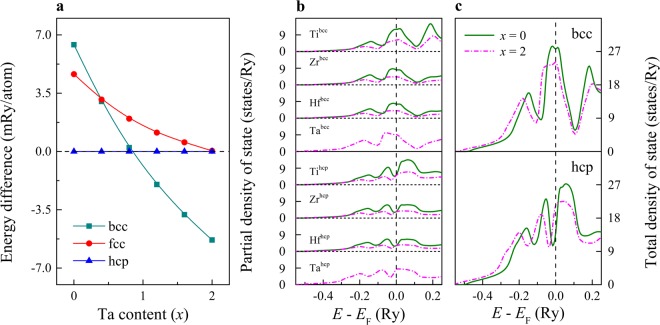
(**a**) The equilibrium total energy of TiZrHfTa_*x*_ as a function of composition for the bcc and fcc structures relative to the hcp structure. The (**b**) partial and (**c**) total density of states for the bcc and hcp phases of TiZrHfTa_*x*_ (*x* = 0 and 2). The position of the Fermi level *E*_F_ is marked by dashed lines.

Figure 2a shows the composition-dependent equilibrium total energies of TiZrHfTa_*x*_ for the bcc and fcc structures relative to that of the hcp structure. It is found that for the equiatomic TiZrHf, the structures follow the hcp, fcc and bcc sequence. The same trend was reported in pure Ti, Zr, and Hf metals^22^. The present theoretical results indicate that the hcp phase is energetically stable in the low-Ta region, and the bcc phase becomes favorable in the upper part of the actual concentration range. At the same time, the fcc phase is always less stable with respect to the hcp phase for all compositions considered here. These theoretical predictions for TiZrHfTa_*x*_ are in line with the experimental observation^19^, and are consistent with the previous theoretical results for the alloy components^23^.

Further insight into the phase stability can be gained by considering the electronic density of state (DOS). In Fig. 2, we present the partial and total DOS of TiZrHfTa_*x*_ for the bcc and hcp structures, respectively. The zero of the energy axis is at the Fermi level (*E*_F_). It is clear that for the equiatomic TiZrHf, the bottom of the pseudo-gap is located near *E*_F_ in the hcp phase, whereas *E*_F_ lies in a region of high DOS in the bcc phase. This implies that the hcp phase has smaller kinetic energy and thus it is more stable than the bcc phase. From Fig. 2c (upper panel), we find that *E*_F_ is shifted towards the upper part of the bcc peak with increasing *x*, which is due to the more d-valence electrons of Ta as compared to the other alloy components. On the other hand, the small decrease of the bcc DOS near *E*_F_ is likely to be associated with the decrease of the hcp-stabilizing elements and the addition of Ta which in pure bcc phase has a pseudo-gap slightly above *E*_F_. The pseudo-gap is clearly visible in the bcc partial DOS of alloy components as shown in Fig. 2b (upper four panels). However, due to the hybridization with the so-called hcp-type TiZrHf matrix, Ta loses electrons and thus *E*_F_ is shifted towards the peak below the bcc pseudo-gap. In contrast, as shown in Fig. 2b (lower four panels) and Fig. 2c (lower panel), for the hcp lattice *E*_F_ shifts towards the ascending zone (i.e., towards the peak above the hcp pseudo-gap in the Ta-free alloy) when Ta is added, indicating a clear destabilization of the hcp phase. This explains the relative stability of the bcc phase over hcp with increasing Ta content. We notice that the steep negative DOS slope around *E*_F_ in high-Ta system (not shown) is similar to that of pure V and Nb metals^24–26^. Namely, anomalous temperature dependence may also occur in these HEAs, which could be verified by further theoretical and experimental analysis.

**Figure 3:**
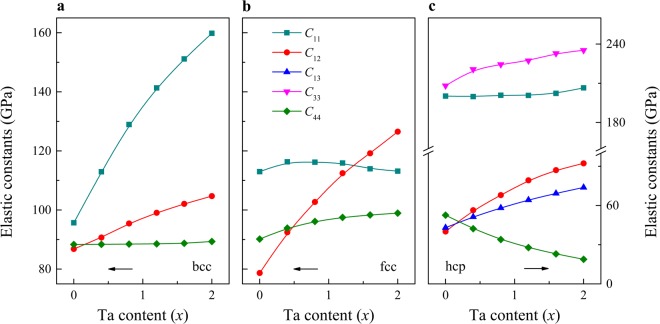
The complete set of independent single-crystal elastic constants of TiZrHfTa_*x*_ as a function of composition for the (**a**) bcc, (**b**) fcc, and (**c**) hcp structures.

The mechanical stability criteria can be formulated in terms of single-crystal elastic constants. In Fig. 3, we present the complete set of elastic constants of TiZrHfTa_*x*_ for the bcc, fcc and hcp structures, respectively. It is found that in the high-Ta region, the fcc phase loses mechanical stability, as one of the stability criteria for cubic crystal, namely ${C}_{11} > |{C}_{12}|$, is broken, but the other two restrictions, ${C}_{44} > 0$ and ${C}_{11}+2{C}_{12} > 0$, remain valid over the whole composition range. On the other hand, the bcc phase always satisfies the above conditions. The hcp phase is also predicted to be mechanically stable, since the requirements^27^ ${C}_{11} > |{C}_{12}|$, ${C}_{44} > 0$, ${C}_{11}{C}_{33} > {C}_{13}^{2}$ and ${C}_{33}({C}_{11}+{C}_{12}) > 2{C}_{13}^{2}$ are fulfilled for all compositions. Taking into account the mechanical stability results and those found for the thermodynamic stability, below we discuss the elastic behavior of TiZrHfTa_*x*_ in the bcc and hcp phases only.

For the bcc phase of TiZrHfTa_*x*_, as shown in Fig. 3a, ${C}_{11}$ and ${C}_{12}$ increase almost linearly with increasing Ta content, while ${C}_{44}$ is much less sensitive to the change of the composition. The three cubic elastic constants calculated for the equiatomic TiZrHfTa are ${C}_{11}$ = 135.6 GPa, ${C}_{12}$ = 97.4 GPa, ${C}_{44}$ = 88.6 GPa, which yield 19.1 GPa for the tetragonal elastic constant $C^{\prime} =({C}_{11}-{C}_{12})/2$. For reference, pure Ta in the bcc phase has $C^{\prime}$ around 53 GPa^28^. Hence, this alloys is predicted to show much weaker dynamical stability against tetragonal deformation as compared to pure Ta. With decreasing Ta, the bcc phase is destabilized as $C^{\prime}$ decreases. On the other hand, for the hcp phase (Fig. 3c), we find that ${C}_{11}$, ${C}_{12}$, ${C}_{13}$ and ${C}_{33}$ decrease with decreasing Ta content whereas ${C}_{44}$ and ${C}_{66}=({C}_{11}-{C}_{12})/2$ remarkably increase. This feature confirms the stabilization of the hcp phase in the low-Ta region.

**Figure 4:**
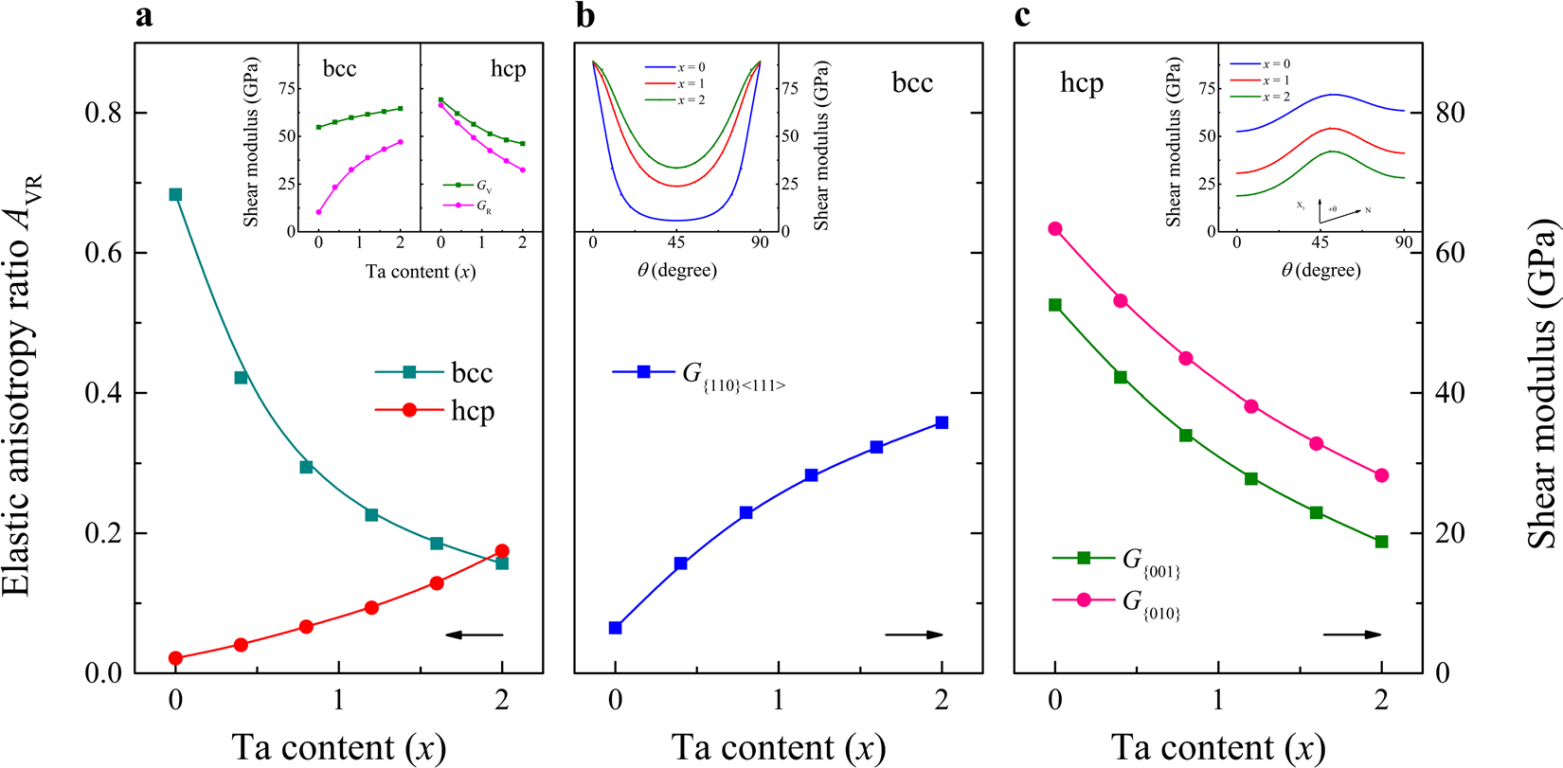
(**a**) The elastic anisotropy *A*_VR_ for the bcc and hcp phases of TiZrHfTa_*x*_ as a function of composition. Inset shows the alloying-induced changes of the Voigt and Reuss shear modulus. (**b**) The single-crystal shear elastic modulus associated with the {110}〈111〉 slip system for the bcc phase of TiZrHfTa_*x*_ as a function of composition. Inset shows the orientation dependence of the shear modulus in the first quadrants of the {110} plane for *x* = 0, 1, and 2. (**c**) The average shear modulus of the basal and primary prismatic planes for the hcp phase of TiZrHfTa_*x*_ as a function of composition. Inset shows the angular variation of the shear modulus for *x* = 0, 1, and 2.

Using the single-crystal elastic constants, one can estimate the elastic properties of polycrystalline alloys. Figure 4a shows the ratio ${A}_{{\rm{VR}}}=({G}_{{\rm{V}}}-{G}_{{\rm{R}}})/({G}_{{\rm{V}}}+{G}_{{\rm{R}}})$ of TiZrHfTa_*x*_ for the bcc and hcp structures, where ${G}_{{\rm{V}}}$ and ${G}_{{\rm{R}}}$ are the Voigt and Reuss bounds for the shear modulus, respectively^29^. We mention that *A*_VR_ is independent of the crystal structure, and thus it can be used as a measure of the elastic anisotropy of various structures (for isotropic crystals *A*_VR_ is zero). It is found that *A*_VR_ increases from 0.02 at *x* = 0 to 0.17 at *x* = 2 in the hcp phase, whereas in the bcc phase it varies from 0.68 to 0.16 when *x* changes from 0 to 2. Previous work reported that most of the cubic and low symmetry crystals have *A*_VR_ below 0.21^20,27,30^. On this scale, the elastic anisotropy of hcp TiZrHfTa_*x*_ can be considered small, however, the bcc elastic anisotropy in the low-Ta region is large, which indicates large uncertainties in the predicted average bcc shear modulus. In the following, we make use of the particular shear moduli defined for selected crystal planes.

We consider the $\{110\}\langle 111\rangle$ slip system for the bcc lattice due to its special role in the plastic deformation. The associated shear modulus can be written as $G\{110\}\langle 111\rangle =3{C}^{{\rm{^{\prime} }}}{C}_{44}/({C}^{{\rm{^{\prime} }}}+2{C}_{44})$. We notice that this modulus in fact expresses the shear for any possible shear plane $\{lmn\}$ which contains the 〈111〉 shear direction. Figure 4b shows the alloying-induced changes of $G\{110\}\langle 111\rangle$ of TiZrHfTa_*x*_ for the bcc structure, and the inset shows the orientation dependence of the shear modulus in the first quadrants of the $\{110\}$ plane^31^. The shear modulus in the $\{110\}$ plane is found to decrease with decreasing Ta for all directions. Meanwhile, there are clear differences between various directions, which emerge for highly anisotropic systems (${A}_{{\rm{Z}}}={C}_{44}/C^{\prime}  > 3$) as indicated from Fig. 3a. Furthermore, the equiatomic bcc TiZrHfTa has $G\{110\}\langle 111\rangle$ = 25.9 GPa, which is about four times larger than that of the Ta-free alloy. The corresponding experimental value for pure bcc Ta is around 60.2 GPa^28^. The sharp decrease of the shear modulus is a consequence of the decreased mechanical stability marked by the change of $C^{\prime}$ (Fig. 3a). It is known that the shear modulus represents the resistance to reversible deformations upon shear stress, and the reduction of Ta substantially softens the elastic modulus associated with the $\{110\}\langle 111\rangle$ shear. This elastic softening promotes dislocation mobility and thus it is crucial for the ductile behavior.

Considering the relatively low elastic anisotropy of hcp TiZrHfTa_*x*_, in Fig. 4c, we plot the average shear modulus on the basal and primary prismatic planes as a function of Ta content. The inset of Fig. 4c presents the effect of differently oriented planes on the values of shear modulus in hexagonal crystals^32^. As a general observation, all of the alloys considered here show a pronounced minimum value of shear modulus on the $\{001\}$ basal plane, for which *θ* is zero, and a tendency to exhibit a maximum between 0° < *θ* < 90°. This behavior is similar to that of some pure hcp metals like Mg, Sc, and Zr^32^. Moreover, the shear modulus of TiZrHfTa_*x*_ varies with the amount of Ta from ~52–72 GPa at *x* = 0, to ~31–55 GPa at *x* = 1 and to ~18–43 GPa at *x* = 2. The trends are almost parallel to each other, and thus a direct comparison of alloying-induced changes may be appropriate. Particularly, it is clear from Fig. 4c that the shear modulus increases when Ta is reduced, in contrast to the trend obtained for the bcc phase (Fig. 4b). This suggests that the appearance of the hcp crystallites (in the low-Ta region) should promote strengthening. We mention that the bulk moduli in the bcc and hcp phases are close to each other for all considered compositions (not shown), and thus the markedly different shear moduli control the ductile-brittle behavior of the present alloys.

In summary, we put forward a systematic study of the phase stability and elastic behavior of the refractory TiZrHfTa_*x*_ (0 ≤ *x *≤ 2) HEAs by using first-principle alloy theory. The total-energy and electronic structure calculations predict that the hcp phase remains stable in the low-Ta region, whereas Ta addition makes the bcc phase thermodynamically favorable. This trend is in line with the experimental observation. Analysis of the elastic parameters of the bcc phase shows that a relatively soft shear system $\{110\}\langle 111\rangle$ exists, and the resolved shear modulus decrease rapidly when Ta is gradually reduced, which plays an important role in the enhanced ductility of the dual-phase system. On the other hand, the hcp phase turns out to be elastically nearly isotropic, and the average shear modulus increases with decreasing Ta concentration, which implies that the emerging hcp precipitates promotes strengthening. We propose that the significantly different elastic behavior of the considered alloy phases can serve as a guide for the development of multi-phase HEAs with adjustable mechanical performance.

## Methods

The present *ab initio* calculations were based on the exact muffin-tin orbitals (EMTO) method^20^, in combination with the coherent potential approximation (CPA)^33–35^. Details about the EMTO-CPA approach and its self-consistent implementation can be found in previous work^20^. The one-electron Kohn-Shan equations were solved within the scalar-relativistic approximation and soft-core scheme. The muffin-tin basis set included *s*, *p*, *d* and *f* orbitals. The exchange-correlation effects were treated within the generalized gradient approximation in the form of Perdew-Burke-Ernzerhof (PBE)^36^. The equilibrium volume and bulk modulus were extracted from the equation of state fitted to the *ab initio* total energies for a series of different volumes. The single-crystal elastic constants were obtained by straining the lattice and evaluating the energy changes due to the strain as a function of its magnitude at equilibrium volume^20,37,38^. All calculations were performed for static lattice and for completely disordered solid solutions. The chemical disorder for both lattices was treated by CPA. The present approach was proved to have the necessary accuracy and predictive power to reveal various parameters and mechanisms for complex multicomponent alloys^39–45^.
